# Research progress on peptides that inhibit melanin synthesis

**DOI:** 10.3389/fphar.2025.1610623

**Published:** 2025-07-02

**Authors:** Binrui Yu, Nailong Wang, Shanshan Cai, Hao Yan, Shaojia Sun, Siru Wang, Ye Li, Zhengting Liang

**Affiliations:** ^1^ College of Pharmacy, Xinjiang Medical University, Urumqi, China; ^2^ College of Traditional Chinese Medicine, Xinjiang Medical University, Urumqi, China; ^3^ Division of Biomedical and Life Sciences, Faculty of Health and Medicine, Lancaster University, Lancaster, United Kingdom; ^4^ Institute of Biopharmaceutical and Health Engineering, Tsinghua Shenzhen International Graduate School, Shenzhen, China; ^5^ College of Public Health, Xinjiang Medical University, Urumqi, China; ^6^ College of Bioengineering, Sichuan University of Science and Engineering, Yibin, China

**Keywords:** melanin, excessive deposition, inhibition, tyrosinase, whitening, bioactive peptides

## Abstract

Melanin produced by melanocytes, primarily determines human skin color and protects against ultraviolet radiation. However, excessive melanin deposition can lead to skin conditions such as freckles, age spots, and moles, potentially causing aesthetic concerns and psychological distress. Consequently, there is significant research interest in developing safe and effective whitening products that inhibit melanin synthesis. Bioactive peptides represent a promising compound category that effectively reduces melanin synthesis with minimal side effects. This review explores melanin pigmentation, identifies sources of peptides that inhibit melanin synthesis, and elucidates the mechanisms by which these peptides operate, aiming to contribute to developing novel whitening products.

## 1 Introduction

Melanin is the primary determinant of human skin color and is a biological pigment widely found in animals, plants, and microorganisms (Jung et al., 2015; Sugumaran and Barek, 2016). It is a complex polymer formed by indole or phenolic compounds synthesized within melanosomes-specialized organelles in melanocytes (Li R. et al., 2024; Snyman et al., 2024). Melanosomes are lysosomal organelles (Bissig et al., 2016) that house various enzymes essential for melanin synthesis, including tyrosinase (TYR), which is the rate-limiting enzyme in this process (Aizawa and Yamamuro, 2024). Melanocytes produce two types of melanin: pheomelanin and eumelanin (Le et al., 2021; Boo, 2022; Bento-Lopes et al., 2023; Qiu et al., 2023).

Melanin is a crucial pigment in the human body, influencing the color of human skin, hair, and eyes (Wang et al., 2024). It protects against harmful ultraviolet rays, reducing skin damage (Galibert et al., 2001; Mueller and Neuhauss, 2014; Solano, 2020; Onoda et al., 2024). However, excessive melanin deposition can lead to aesthetic concerns and health issues, such as freckles, moles, melanoma, and senile plaques (Azimi et al., 2024). Additionally, there is growing evidence linking pigmentation disorders with neurodegenerative diseases, including Parkinson’s disease and Alzheimer’s disease (Tseng et al., 2015; Berg and Berg, 2023).

Recent research into melanin and inhibitors of melanin synthesis has grown exponentially. Various melanin synthesis inhibitors have been developed, offering potential treatments for diseases linked to excessive melanin deposition (Baber et al., 2023; Lu et al., 2023; Veerichetty and Saravanabavan, 2023). Amongst these inhibitors, bioactive peptides have emerged as a significant area of interest due to their efficacy in reducing melanin synthesis with minimal side effects (Errante et al., 2024). In this review, we explore the biosynthesis and regulation of melanin, detail the role of bioactive peptides as melanin synthesis inhibitors, and discuss the mechanisms by which these peptides function, all aimed at developing new whitening products.

## 2 Biosynthesis of melanin and its regulation

### 2.1 Melanin synthesis pathway

The specific process of melanin synthesis begins with the conversion of tyrosine into L-Dopa catalysed by tyrosinase, the central glycoprotease in the melanosome region and the only rate-limiting enzyme in this process (Kim et al., 2023). L-Dopa is oxidized to dopaquinone, an active intermediate and crucial precursor for converting melanin into eumelanin and brownish melanin (Olivares et al., 2001). When the ratio of dopaquinone to cysteine is low, L-dopaquinone (L-DQ) undergoes self-cyclization and oxidation to form dopachrome (DC). With the aid of tyrosinase-related protein 2, dopachrome is then converted into 5,6-dihydroxyindole-2-carboxylic acid (DHICA), and 5,6-dihydroxyindole (DHI) (Boissy et al., 1998; Olivares et al., 2001). These intermediates eventually combine within melanosomes to form eumelanin.

**FIGURE 1:**
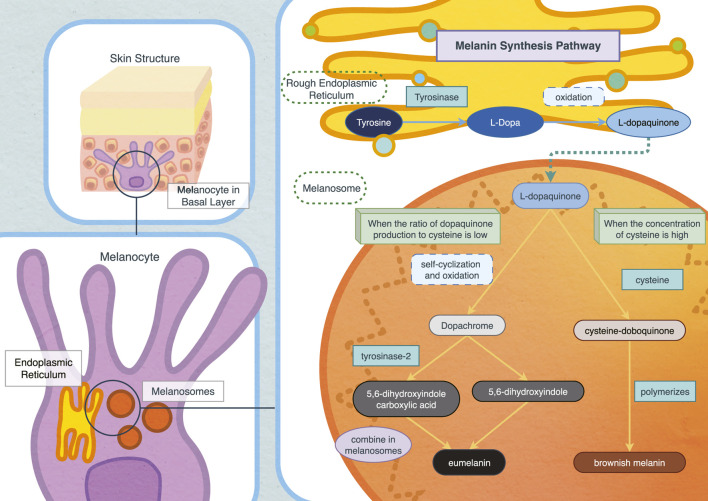
Biosynthesis pathway of melanin *in vivo*.

In conditions where cysteine concentration is high, red-brown melanin is produced. Doboquinone reacts with cysteine to form cysteinyldopa, which further polymerizes to produce brownish melanin (Galvan and Solano, 2016; Wakamatsu et al., 2017). Microphthalmia-related transcription factor (MITF) regulates the expression of tyrosinase and is a primary target for influencing melanin synthesis (Ikarashi et al., 2020; Yu et al., 2021). Phosphorylation of MITF can enhance melanin synthesis (Joyjamras et al., 2022). Moreover, studies have indicated that nicotinamide nucleotide transhydrogenase (NNT) inhibits melanin synthesis by regulating oxidative stress, presenting an alternative to the common TYR pathway and MITF pathway and highlighting the role of the reactive oxygen species (ROS) pathway controlling melanin synthesis (Allouche et al., 2021; Park et al., 2022; Figure 1).

### 2.2 Regulation of pigmentation

Melanin pigmentation is a crucial defense mechanism, protecting humans from external hazards, particularly ultraviolet (UV) rays. It also plays a significant role in the body’s immune response and metabolism, safeguarding against harmful substances (Mueller and Neuhauss, 2014; Koike and Yamasaki, 2020). Pigmentation disorders are the third most common reason for dermatological consultations (Cestari et al., 2014). The primary causes of skin pigmentation disorder include inflammation, ultraviolet radiation, and interactions between specific diseases and medications. This review will further explore the causes of pigmentation, examining the impacts of ultraviolet exposure, inflammation, and genetic inheritance.

#### 2.2.1 Ultraviolet exposure

UV exposure is critical to skin pigmentation (Al-Sadek and Yusuf, 2024). Studies have demonstrated that UV radiation directly stimulates melanocytes, enhancing their melanin secretion. Specifically, UV irradiation increases the release of melanin-inducing factors such as stem cell factor (SCF), endothelin-1 (ET-1), and Pro-opiomelanocortin (POMC). POMC is enzymatically hydrolyzed into α-melanocyte-stimulating hormone (α-MSH) and adrenocorticotropic hormone, where α-MSH binding stimulates eumelanin production (Koike and Yamasaki, 2020; Zhang et al., 2024b).

Ultraviolet A (UVA), with a wavelength of 320 nm–400 nm, has the longest wavelengths in the UV spectrum and can easily penetrate skin tissue (Al-Sadek and Yusuf, 2024). UVA’s strong penetration ability and DNA’s weak absorption capacity for UVA can lead to DNA damage through photosensitive reaction (Agar et al., 2004; D'Orazio et al., 2013). Moreover, the absorption of UVA by the skin generates ROS, inducing oxidative stress, which in turn promotes melanosis (Allouche et al., 2021; Bernerd et al., 2022).

Exposure to Ultraviolet B (UVB), known for causing sunburn, is a significant external factor influencing melanin production (Zhang et al., 2024b). Prolonged UVB exposure activates melanocytes, increasing melanin content, which accelerates pigmentation and potentially contributes to various skin disease such as freckles (Azimi et al., 2024).

#### 2.2.2 Genetic inheritance or inflammation

Pigmentation disorders can result from various factors, including inflammation and genetic inheritance (Hossain et al., 2021; Tearle et al., 2025). Studies have shown that several inflammatory factors are involved in skin pigmentation (Feng et al., 2025). Interleukin-1 (IL-1) a key player in cellular inflammatory responses, and exists in two forms: IL-1α and IL-1β. Keratinocyte growth factor (KGF) facilitates the transport of melanosomes, and when combined with IL-1α, it promotes pigmentation (Cardinali et al., 2005; Chen et al., 2010). IL-1β has been shown to upregulate the expression of tyrosinase and tyrosine-related protein-1 (TRP1), potentially enhancing skin pigmentation by increasing melanin gene expression and promoting the production of additional inflammatory factors (Yang et al., 2022).

IL-4, through the JAK2-STAT6 pathway, reduces MITF and TRP-1 expression in normal human melanocytes (NHM), thereby inhibiting melanin production (Choi et al., 2013). IL-13, which shares similar receptor structures and signaling pathways with IL-4, is also involved in melanogenesis regulation. Epidermal γδ T cells produce both IL-4 and IL-13, with IL-13 being the more abundant cytokine (Renauld, 2001; Nishimura et al., 2008; Kim et al., 2014). Ginsenoside F1 treatment increases IL-13 output from these T cells, contributing to whitening by inhibiting tyrosinase and DCT expression. IL-13 may directly regulate melanogenesis through the JAK2-STAT6 signaling pathway (Han et al., 2014).

While IL-17 alone does not inhibit pigmentation, it significantly amplifies the inhibitory effect of TNF on melanin synthesis through a synergistic interaction (Wang et al., 2013). IL-18 enhances primary human melanocyte growth by inactivating PTEN via the AKT/NF-κB pathway (Zhou et al., 2013; Zhou et al., 2016). IL-33 has been shown to stimulate melanin biosynthesis in NHEM by promoting phosphorylation of p38 MAPK and CREB, leading to increased expression of TYR, TRP-1, and DCT through MITF, ultimately resulting in increased melanin production (Zhou et al., 2014). COX-2 may contribute to the formation of chloasma by activating tyrosinase and melanogenesis-related molecules, therefore promoting melanogenesis (Rodriguez-Arambula et al., 2015).

Gene factors also play a crucial role in inducing pigmentation. For example, differential expression of genes such as SLC24A5 and SLC45A2 influences variations difference in skin color (Soejima and Koda, 2007; Haltaufderhyde and Oancea, 2014).

### 2.3 Traditional products used to inhibit melanin synthesis

The potential of products that inhibit melanin synthesis, which can have a whitening effect, has long attracted attention. Vitamin C (VC), a well-known free radical scavenger, is the most commonly used, as it effectively neutralizes free radicals caused by oxidative stress from UV irradiation (Gelmi et al., 2022). Other products, such as Vitamin E (VE) and gentisic acid, also serve similar functions (Miao et al., 2019; Sandhu et al., 2023). VC is an acidic compound inhibiting tyrosinase activity through cytoplasm acidification, suppressing melanin synthesis (Nam et al., 2023). As tyrosinase is the key enzyme regulating melanin synthesis, tyrosinase inhibitors are often utilized to inhibit melanin production (Pillaiyar et al., 2017). Natural tyrosinase inhibitors include flavonoids, hydroquinone and its derivatives, stilbene, chalcone, arbutin, kojic acid and coumarin, and some newly synthesized inhibitors (Lee et al., 2016).

While these tyrosinase inhibitors are frequently used in whitening products, studies have highlighted significant side effects (Pillaiyar et al., 2017). For example, long-term use of kojic acid has been shown to have cytotoxic effects, and its instability (due to sensitivity to light, heat, and metal ions) limits its storage and efficacy (Schurink et al., 2007). Hydroquinone use has been associated with several adverse reactions, including allergic dermatitis and depigmentation (Jow and Hantash, 2014). Aminobenzoic acid has also been linked to various side effects, such as headaches, abdominal pain, nausea, and vomiting (Bala et al., 2018; Kim and Lim, 2023).

## 3 Peptides are emerging products that inhibit melanin synthesis with little side effect

Numerous studies have identified bioactive peptides as having various benefits, including antibacterial, antiviral, diabetic, and anticancer properties (Kumar et al., 2024; Nikitovic et al., 2024; Zhang S. et al., 2024; Zhao and Song, 2024). Additionally, many bioactive peptides with anti-melanin synthesis properties have been discovered. These peptides have garnered significant interest from researchers worldwide, due to their potential for development into commercial whitening products, and their inherent advantages of low toxicity, low immunogenicity, and high biocompatibility (Lombardi et al., 2019; Guo et al., 2024).

### 3.1 Melanin-synthesis-inhibiting peptides of anaimal origins

#### 3.1.1 Melanin synthesis-inhibiting peptides from terrestrial animal resources

##### 3.1.1.1 From egg

Phosvitin, a highly phosphorylated protein in egg yolk, contains specific amino acids, with serine accounting for 30% of its composition (Ishikawa et al., 2007; Jie et al., 2018). The unique structure of phosphotoxic protein makes it a potent metal-chelating agent (Okajima et al., 2019; Song et al., 2023). Studies have shown that compounds with metal chelating abilites can also act as melanogenesis inhibitors (Kubo and Kinst-Hori, 1999). In this study, phosphotoxic protein inhibited melanin synthesis by downregulating the expression of MITF, TRP-1, TRP-2, and tyrosinase in B16F10 cells and reducing cAMP levels (Jung et al., 2012). Additionally, phosphotoxic peptide phosphopeptides (PPPs) derived from phosphotoxic protein inhibited α-MSH-induced melanin production in B16F10 melanoma cells. Inhibition rates exceede 30% at a concentration of 3 mg/mL, demonstrating a strong inhibitory effect on tyrosinase activity (Lee et al., 2023).

A 2020 study utilized pepsin and trypsin to hydrolyze egg whites, yielding a hydrolysate with strong monophenolase and diphenolase inhibitory activities (Yap and Gan, 2020). Seven peptides were identified as potential inhibitors of melanin synthesis: ILELPFASGDLLML, GYSLGNWVCAAK, YFGYTGALRCLV, HIATNAVLFFGR, FMMFESQNKDLLFK, SGALHCLK and YFGYTGALR (Yap and Gan, 2020).

Additionally, a 2022 study demonstrated that GYSLGNWVCAAK and CEWHdi both reduced intracellular cAMP levels. While GYSLGNWVCAAK inhibited tyrosinase expression, CEWHdi and CEWHmono suppressed the mRNA expressions of Mitf, Tyr, Trp-1, and Trp-2 (Yap et al., 2023).

##### 3.1.1.2 From bee

Many bioactive substances inhibit melanin synthesis by inhibiting the growth of melanoma cells. Bee venom and melittin, a bee peptide, has been shown to inhibit melanoma cell growth and migration (Kim et al., 2018; Lim et al., 2019). Melittin, a 26-amino acid peptide (NH2-GIGAVLKVLTTGLPALISWIKRKRQQ-CONH2) works in conjunction with bee venom to inhibit the growth of PI3K/AKT/mTOR and MAPK pathways (Lee and Bae, 2016; Rady et al., 2017; Lim et al., 2019). Melittin decreases the total protein content of the key signal molecules, and its inhibitory effect on melanoma is stronger than bee venom alone. Furthermore, studies indicate that inhibition of the PI3K/AKT/mTOR pathway enhances melanoma cell sensitivity to the chemotherapy drug temozolomide (TMZ), suggesting that combining bee venom with TMZ could lead to a more potent inhibitory effect on melanoma (Sinnberg et al., 2009; Niessner et al., 2017).

##### 3.1.1.3 From milk

β-lactoglobulin (BLG), a major milk protein component, has inhibited tyrosinase activity (Mayerhofer et al., 2022). A study in Japan demonstrated that at a concentration of 1 mg/mL, BLG reduced pigmentation in human melanocytes treated with retinol, resulting in only weak pigmentation. However, it remains unclear whether the inhibitory effect was due to the entire BLG protein or a specific component of it.

Subsequent research in Japan found that *Lactobacillus helveticus*-Fermented Milk Whey (LHMW) could inhibit melanin synthesis (Ikarashi et al., 2020). LHMW inhibited an α-MSH-induced increase in tyrosinase, TRP1, and DCT expression at both protein and mRNA levels in mouse B16 melanoma cells. Moreover, the expression of MITF was decreased when LHMW was applied alone. While LHMW has shown the potential to inhibit melanin synthesis, the precise active components responsible for this effect are still under investigation. Given the rich peptides and protein, content of whey and the strong proteolytic activity of *Lactobacillus helveticus*, it is speculated that the peptide in LHWN decreased the expression of MITF (Genay et al., 2009; Ikarashi et al., 2020; Irazoqui et al., 2024; Rackerby et al., 2024).

Additionally, studies from South Korea have indicated that bioconverted fermented milk (BCFM) can prevent melanin production by inhibiting the expression of MITF in B16F1 cells induced by α-MSH. It is suspected that peptides within BCFM are responsible for this activity, although the exact peptides involved remain unidentified (Choi et al., 2024).

##### 3.1.1.4 From silkworm

Silk has two main proteins: sericin and fibroin (Jiang et al., 2024; Noda et al., 2024). Most sericin is discarded during processing, accounting for 20%–30% of a cocoon’s quality (Kato et al., 1998; Wu et al., 2008). A study in Japan demonstrated that sericin inhibits tyrosinase activity in silk. The anti-tyrosinase activity of sericin hydrolysate is thought to be linked to copper chelating properties and high serine content (Wu et al., 2008). The relationship between copper chelating activity and tyrosinase inhibition is well established (Cardoso et al., 2021; Jung et al., 2024).

#### 3.1.2 Melanin-synthesis-inhibiting peptides of aquatic origins

##### 3.1.2.1 From fish

Fish is rich in protein, with content ranging from 10% to 25% (Borges et al., 2023; Honrado et al., 2024). Fish by-products constitute about 60% of the total weight of fish and are often considered low-value products. Many by-products, including fish scales and heads, are underutilized or discarded (Ramakrishnan et al., 2023; Shekoohi et al., 2024). Tilapia scale peptides, prepared through enzymolysis and elution, have strong tyrosinase inhibition *in vitro*. At a 5 mg/mL concentration, the tyrosinase inhibition rate of tilapia scale reached 59.73%, which is significantly higher than the traditional tyrosinase inhibitor arbutin at the same concentration. Furthermore, tilapia scale peptide has demonstrated copper-chelating ability, making it an effective tyrosinase inhibitor (Ju et al., 2022).

In a study conducted in Jiangxi, China, grass carp scale gelatin hydrolyzed by alkaline protease also exhibited significant tyrosinase inhibitory activity. When the fish scale gelatin hydrolysate was used at 5 mg/mL, the tyrosinase inhibition rate was 61.7%. Four new peptides were identified from the hydrolysate using a rapid screening method for tyrosinase inhibitory peptides (TYIPs) via bio-affinity ultrafiltration combined with LC-Orbitrap-MS/MS. DLGFLARGF exhibited high tyrosinase inhibitory activity, with an IC50 value of 3.09 mM. At a 1.6 mg/mL concentration, DLGFLARGF reduced tyrosinase activity by 76.81%, decreasing melanin content (Hu et al., 2022a).

A recent study in South Korea isolated and identified decapeptide (DP, KGYSSYICDK) from the hydrolysate of a *Chromis Notate* by-product. DP exhibited high antioxidant activity, comparable to or greater than VC in FRAP and ABTS assays. It also inhibited tyrosinase activity and reduced melanin synthesis in α-MSH-induced B16F10 cells in a dose-dependent manner. DP showed strong binding to various tyrosinase residues, reducing the mRNA expression of MITF, tyrosinase, and MC1R (Lee et al., 2024).

Additionally, a peptide T-6(FGFRSP), isolated from TFMH, was studied for its interaction with tyrosinase. Molecular docking revealed that T-6 binds within the TYR cavity, forming hydrogen bonds with Val248, Ala323, Asn320, and Asn81, ionic bonds with Glu322, and pi-pi stacking interactions with Phe192. T-6 may inhibit tyrosinase activity and melanin synthesis by binding to the enzyme’s first and second regions (Hu et al., 2024).

##### 3.1.2.2 From shellfish

Shellfish, like fish, is a rich source of high-quality protein and bioactive peptides (Phadke et al., 2021). Meng et al. used alkaline protease from *Bacillus licheniformis* to hydrolyze *Pinctada martensii*, yielding 401 peptides with tyrosinase inhibitory activity. These were identified through ultrafiltration and purification. After amino acid sequence identification and molecular docking, three peptides with the lowest binding energy were obtained: WDRPKDDGGSPIK(W1), DRGYPPVMF(W2) and SGGGGGGGLGSGGSIRSSY(W3). At 1–5 mg/mL concentrations, W3 inhibited melanin synthesis by competitively inhibiting tyrosinase activity. Importantly, W3 demonstrated value without affecting the survival of B16F10 cells (Meng et al., 2023).

Additionally, studies have shown that oyster hydrolysate (OH) inhibits melanin synthesis in B16F10 cells by downregulating the cAMP pathway. In C57BL/6J mice treated with OH, both melanin content and the number of melanoma cells were reduced. The sequence of the OH peptide is Ser-Ser-ASP-ASN-ASN-ASP-Glu-Ala-Lys, with a molecular weight of 1036.39 Da (Han et al., 2019).

A 2023 study in Thailand used computer simulation technology to predict eight candidate peptides for tyrosinase inhibition in abalone peptides: TIP1, TIP2, KNN1, KNN2, KNN3, RF1, RF2, and RF3. These peptides showed no cytotoxic effect on mouse melanoma cells. According to AnOxPePred, TIP2, and KNN1 exhibited the best free radical scavenging and ion-chelating activities. At a concentration of 70.83 μM, KNN1 reduced tyrosinase activity in mushrooms by 50% (Kongsompong et al., 2023).

#### 3.1.3 Peptides from amphibians with melanin synthesis-inhibiting activities

Amphibians produce various peptides with diverse structures and functions, many of which have been studied for their antibacterial, antioxidant, and skin wound-healing properties (McMillan and Coombs, 2020; Feng G. et al., 2021; Wang X. et al., 2023). Research on *Andrias davidianus* (Chinese giant salamander) has gained attention due to its high protein content, which can produce a range of bioactive peptides (Chen et al., 2021; Guo et al., 2023). Among these peptides, antioxidants have been extensively studied, with some also exhibiting melanin-inhibiting properties (Speeckaert et al., 2023; Zhan et al., 2024). However, research on the anti-melanin effects of *Andrias davidianus* remains limited, with more studies focused on *Odorrana andersonii*.

A study in China in 2023 reported the first findings on OA-VI12, a peptide derived from *Odorrana andersonii* which regulates melanin synthesis. OA-VI12 was found to inhibit melanin synthesis in B16 cells. At a concentration of 5 μM, the inhibitory effect of OA-VI12 on melanin production was comparable to that of arbutin, but OA-VI12 showed a significantly stronger inhibitory effect at this concentration. OA-VI12 was shown to promote the expression of miR-122-5p while downregulating the expression of MITF and TYR, suggesting that it inhibited melanin synthesis through the miR-122-5p/MITF/TYR axis. Furthermore, OA-VI12 demonstrated transdermal penetration, inhibiting UVB-induced pigmentation in mouse ears and significantly reducing pigmentation (Wang J. et al., 2023).

Subsequently, another melanin-inhibitory peptide from *Odorrana andersonii,* (Nigrocin-OA27), was identified. Although its molecular weight is twice that of OA-VI12, Nigrocin-OA27 also exhibited a strong transdermal effect. It decreased tyrosinase activity in B16 cells, inhibited melanin synthesis, and interacted with the catalytic site, preventing its binding with L-Dopa, and thereby reducing the enzyme’s catalytic activity (Li J. et al., 2024) (Table 1).

**TABLE 1:** Melanin synthesis inhibitory peptides from anaimal origins.

Compound name	Peptide sequence	Source	Mechanism	IC50	References
Phosvitin phosphopeptides	Unknown	Egg	Inhibit the activity of mushroom tyrosinase; Inhibit cell tyrosinase activity and MITF expression	Unknown	Lee et al. (2023)
CEWHdi	Unknown	Chicken egg white	Decrease cAMP level and inhibit cell tyrosinase expression	3.04 mM	Yap et al. (2023)
CEWHmono		Chicken egg white	inhibit cell tyrosinase expression		Yap et al. (2023)
Unnamed	ILELPFASGDLLML	Chicken egg white	Presumably, inhibits monophenolase and diphenolase	Unknown	Yap and Gan, (2020)
Unnamed	GYSLGNWVCAAK	Chicken egg white	Presumably inhibits monophenolase and diphenolase; inhibits tyrosinase expression and reduces cAMP level	Unknown	Yap and Gan, (2020); Yap et al. (2023)
Unnamed	YFGYTGALRCLV	Chicken egg white	Presumably, inhibits monophenolase and diphenolase	Unknown	Yap and Gan, (2020)
Unnamed	HIATNAVLFFGR	Chicken egg white	Presumably, inhibits monophenolase and diphenolase	Unknown	Yap and Gan, (2020)
Unnamed	FMMFESQNKDLLFK	Chicken egg white	Presumably, inhibit monophenolase and diphenolase	Unknown	Yap and Gan, (2020)
Unnamed	SGALHCLK	Chicken egg white	Presumably, inhibit monophenolase and diphenolase	Unknown	Yap and Gan, (2020)
Unnamed	YFGYTGALR	Chicken egg white	Presumably, inhibit monophenolase and diphenolase	Unknown	Yap and Gan, (2020)
Melittin	GIGAVLKVLTTGLPALISWIKRKRQQ	Bee	Inhibit PI3K/AKT/mTOR and MAPK pathways; inhibit the growth and migration of melanoma cells	Unknown	Lim et al. (2019)
Sericin hydrolysate	Unknown	Silk	Inhibit the activity of mushroom tyrosinase; Chelat ferrous ion	8.71 mg/mL; 0.128 mg/mL	Wu et al. (2008)
Unknown	Unknown	BCFM	Inhibition of melanin synthesis in B16F1 cells; Inhibition of MITF activity in B16F1 cells	Unknown; Unknown	Genay et al. (2009)
Tilapia scale peptides	Unknown	*Oreochromis niloticus*	Chelate copper ions; Inhibition of cell tyrosinase activity	Unknown	Ju et al. (2022)
Unnamed	DLGFLARGF	Grass carp scale gelatin	Inhibition of cell tyrosinase activity	3.09 mM	Hu et al. (2022a)
DP	KGYSSYICDK	Chromis Notate	Inhibition of cell tyrosinase activity	Unknown	Lee et al. (2024)
T-6	FGFRSP	Takifugu flavidus	Inhibit the activity of mushroom tyrosinase; Inhibition of melanin synthesis in melanoma cells	Unknown	Hu et al. (2024)
W1	WDRPKDDGGSPIK	Pinctada martensii	Inhibition of mushroom tyrosinase activity	Unknown	Meng et al. (2023)
W2	DRGYPPVMF	Pinctada martensii	Mixed inhibition of tyrosine monophenolase activity	Unknown	Meng et al. (2023)
W3	SGGGGGGGLGSGGSIRSSY	Pinctada martensii	Competitive inhibition of tyrosine monophenolase activity	3.04 mg/mL	Meng et al. (2023)
OH	Ser-Ser-Asp Asn-Asn-Asp-Glu-Ala-Lys	Oyster	Inhibition of mushroom tyrosinase activity	Unknown	Han et al. (2019)
TIP1	TASSDAWYR	Haliotis diversicolor	Inhibition of cell tyrosinase activity	Unknown	Kubglomsong et al. (2018)
TIP2	SAPFMPDAFFRNV	Haliotis diversicolor	Inhibition of cell tyrosinase activity	Unknown	Kongsompong et al. (2023)
KNN1	NICECMK	Haliotis diversicolor	Inhibition of cell tyrosinase activity	Unknown	Kongsompong et al. (2023)
KNN2	TSQMSRSSSR	Haliotis diversicolor	Inhibition of cell tyrosinase activity	Unknown	Kongsompong et al. (2023)
KNN3	KKNYRVSEAYK	Haliotis diversicolor	Inhibition of cell tyrosinase activity	Unknown	Kongsompong et al. (2023)
RF1	SAPTFFR	Haliotis diversicolor	Inhibition of cell tyrosinase activity	Unknown	Kongsompong et al. (2023)
RF2	NSSLRVQSR	Haliotis diversicolor	Inhibition of cell tyrosinase activity	Unknown	Kongsompong et al. (2023)
RF3	SQSNSRSVSR	Haliotis diversicolor	Inhibition of cell tyrosinase activity	Unknown	Kongsompong et al. (2023)
OA-VI12	VIPFLACRPLGL	Odorrana andersonii	Inhibition of B16 cells and mouse ear melanin synthesis; Inhibition of cell tyrosinase activity	Unknown	Wang J. et al. (2023)
Nigrocin-OA27	‘GFLSKPLPVGRKIVPWLSGLC’	Odorrana andersonii	Inhibition of mushroom tyrosinase; Reduce the content of melanin in B16 cells	229.2 μM	Li J. et al. (2024)

### 3.2 Melanin-synthesis-inhibiting peptides of plant origins

#### 3.2.1 From *pseudostellaria heterophylla*

In a 1994 study in Japan, seven cyclic peptides were extracted from the roots of *Pseudostellaria heterophylla*, all of which exhibited inhibitory activity against mushroom tyrosinase. These peptides were named Pseudostellaria A, B, C, D, E, F, and G (Morita et al., 1994a; Morita et al., 1994b; Morita et al., 1994c). Pseudostellaria C, D and G were found to effectively inhibit melanin synthesis when used to treat melanoma cells cultured *in vitro*.

#### 3.2.2 From rice

Rice protein and rice bran protein hydrolysates are rich in bioactive peptides (Ochiai et al., 2016; Kubglomsong et al., 2018; Zhang et al., 2020). Ruixue Zhang and colleagues used alkaline protease and neutralizing enzymes to hydrolyze rice, and then screened for characteristics, identifying three peptides: LLK, LPK, and PEK. The UVB-induced increase in melanin content and tyrosinase activity was significantly reduced in UVB-irradiated PIG1 cells treated with rice protein hydrolysates. At a 200 μg/mL concentration, both melanin content and tyrosinase activity were lower than those in the positive control group. LLK, LPK, and pEK all reduced melanin content and tyrosinase activity, with PEK exhibiting the strongest inhibitory effect. LLK and pEK downregulated the mRNA expression of TRP-1 and TRP-2 (p < 0.01). These peptides regulate the JNK/β-Trcp/NFκB-p65/MITF signaling pathway at both the mRNA and protein levels to inhibit melanin synthesis (Zhang et al., 2020).

Rice bran protein, a by-product of rice production, has also been explored for its bioactive peptides (Zhang et al., 2024a). Akihito Ochiai isolated three peptides- CT-1, CT-2, and CT-3 from rice bran protein with tyrosinase inhibitory activity. Previous research indicated that TH-10 and P4-peptides with similar sequences-share seven identical amino acid residues. TH-10 contains a tyrosine residue at the N- terminal, whereas P4 has tyrosine residue at the center, N- terminal, and C- terminal. Further analysis of P4 showed that the C-terminal tyrosine residues were the most critical for its tyrosinase inhibitory activity (Shen et al., 2019). CT-1, CT-2, and CT-3 all contained C-terminal tyrosine residues and significantly inhibited tyrosinase activity. CT-1 and CT-3 promoted melanin synthesis in mouse B16 melanoma cells, but CT-2 inhibited melanin synthesis without cytotoxicity (Ochiai et al., 2016). Additionally, researchers found that rice bran albumin exhibited higher tyrosinase inhibitory activity than other protein components. After the hydrolysis of rice bran albumin by papain, 13 peptides were obtained by structural analysis, most of which had characteristics of metal-chelating peptides and tyrosinase inhibitors (Kubglomsong et al., 2018).

#### 3.2.3 From flaxseed

Cyclic peptides derived from flaxseed have been proven to possess anti-inflammatory and anticancer properties (Shim et al., 2022; Shim et al., 2024). Recent studies have also demonstrated that a cyclic peptide mixture from flaxseed exhibits tyrosinase inhibitory activity and can inhibit melanin synthesis in B16F10 cells. The proposed mechanism suggests that this effect occurs through the downregulation of the CREB pathway, thereby inhibiting melanin synthesis (Yoon et al., 2023).

#### 3.2.4 From vigna

Zhiwei Shen and colleagues identified a novel peptide, ECGYF (designated EF-5), from Vigna, which exhibits both tyrosinase inhibitory activity and free radical scavenging ability. In both *in vivo* and *in vitro* experiments, EF-5 demonstated a stronger inhibitory effect on tyrosinase than glutathione and arbutin. Molecular docking studies revealed that hydrogen bond and hydrophobic interaction between EF-5 and tyrosinase residues influence tyrosinase activity. It is suggested that EF-5 may induce a conformational change in tyrosinase, which differs from the mechanism of glutathione (Shen et al., 2019).

#### 3.2.5 From walnut

Feng et al. researched walnuts by hydrolyzing DWMP with alkaline protease and filtering it through an ultrafiltration membrane to obtain DWMPH. The DWMPH was divided into four components based on molecular weight, and the tyrosinase inhibitory activity of each component was evaluated. Interestingly, the results revealed that all four components inhibited both the monophenolase and diphenolase activity of tyrosinase, which had smaller molecular weights, correlating with greater tyrosinase inhibitory activity. Dwmphs-Ⅳ, with the smallest molecular weight, exhibited the highest inhibitory activity, showing ic50 of 3.52 mg/mL and 2.65 mg/mL for monophenolase and diphenolase respectively. Further purification of DWMPHs-Ⅳ yielded three components, with F2 showing the strongest inhibitory effect. Molecular docking studies were performed to assess the binding affinity of these peptides with tyrosinase, where lower scores indicated more stable and reasonable binding. The tripeptide FPY was identified as the most stable compound, with the highest binding affinity. The ic50 values for tyrosinase monophenolase and bisphenolase by FPY were 1.11 mM and 3.20 mm respectively. FPY was found to be a competitive inhibitor of tyrosinase and was not degraded by digestive enzymes (Feng Y.-X. et al., 2021).

#### 3.2.6 From *chaenomeles speciosa*

*Chaenomeles speciosa* is known for its anti-tumor, anti-oxidation, and anti-inflammatory effects, but there is limited research on its anti-melanin synthesis properties (Zhang et al., 2010; Xie et al., 2015). In 2020, Deng et al. hydrolyzed and purified papaya seed proteins, obtained two new peptides: NYRRE (F1-a) and RHAKF(F1-b). RHAKF exhibited stronger DPPH radical scavenging ability and lipid peroxidation abilities in antioxidant activity tests. Molecular docking studies revealed more docking sites between RHAKF and tyrosinase, with a closer binding affinity. The IC values for RHAKF and NYRRE were 8.69 mg/mL and 1.15 mg/mL, respectively. RHAKF may possess an imidazole ring capable of metal chelation (Deng et al., 2020) (Table 2).

**TABLE 2:** Melanin synthesis inhibitory peptides from plant origins.

Compound name	Peptide sequence	Source	Mechanism	IC50	References
PseudostellarinsA	Cyclo [GPYLA]	*Pseudostellaria heterophylla*	Inhibition of mushroom tyrosinase pathway	131 μM	Morita et al. (1994c)
PseudostellarinsB	Cyclo [GIGGGPPF]	*Pseudostellaria heterophylla*	Inhibition of mushroom tyrosinase pathway	187 μM	Morita et al. (1994c)
PseudostellarinsC	Cyclo [GTLPSPFL]	*Pseudostellaria heterophylla*	Inhibition of mushroom tyrosinase pathway; Inhibition of melanin production in B16 melanoma cells	63 μM; 171 μM	Morita et al. (1994c)
PseudostellarinsD	Cyclo [GGYPLIL]	*Pseudostellaria heterophylla*	Inhibition of mushroom tyrosinase pathway; Inhibition of melanin production in B16 melanoma cells	100 μM; 49 μM	Morita et al. (1994c)
PseudostellarinsE	Cyclo [GPPLGPVIF]	*Pseudostellaria heterophylla*	Inhibition of mushroom tyrosinase pathway	175 μM	Morita et al. (1994c)
PseudostellarinsF	Cyclo [GGYLPPLS]	*Pseudostellaria heterophylla*	Inhibition of mushroom tyrosinase pathway	50 μM	Morita et al. (1994c)
PseudostellarinsG	Cyclo [PFSFGPLA]	*Pseudostellaria heterophylla*	Inhibition of mushroom tyrosinase pathway; Inhibition of melanin production in B16 melanoma cells	75 μM; 102 μM	Morita et al. (1994c)
Unnamed	LLK	Rice protein	Decrease the expression of TRP-1 and TRP-2 to affect melanin synthesis	Unknown	Zhang et al. (2020)
Unnamed	LPK	Rice protein	Decrease the expression of TRP-1 and TRP-2 to affect melanin synthesis	Unknown	Zhang et al. (2020)
Unnamed	PEK	Rice protein	Decrease the expression of TRP-1 and TRP-2 to affect melanin synthesis	Unknown	Zhang et al. (2020)
CT-1	HGGEGGRPY	Rice bran protein	Inhibition of mushroom tyrosinase activity	Unknown	Ochiai et al. (2016)
CT-2	LQPSHY	Rice bran protein	Inhibition of mushroom tyrosinase activity; Inhibition of melanin synthesis in melanoma	156 μM; 500 μM	Ochiai et al. (2016)
CT-3	HPTSEVY	Rice bran protein	Inhibition of mushroom tyrosinase activity	Unknown	Ochiai et al. (2016)
TH-10	Mrsresswy	Rice	Inhibition of mushroom tyrosinase activity	102 μM	Shen et al. (2019)
P4	YRSRKYSSWY	Unknown	Inhibition of mushroom tyrosinase activity; Inhibition of tyrosine monophenolase activity in mushrooms	40 μM; 123 μM	Shen et al. (2019), Zhang et al. (2020)
Linosorbs	Unknown	Flaxseed	Inhibit the activity of mushroom tyrosinase; Inhibition of melanin synthesis in B16F10 cells	Unknown; Unknown	Yoon et al. (2023)
EF-5	ECGYF	Vinga	Inhibition of mushroom tyrosinase activity; Inhibition of melanin synthesis in A375 cells	0.46 mM; Unknown	Shen et al. (2019)
Unnamed	FPY	Walnut	Inhibition of tyrosine monophenolase activity in mushrooms; Inhibition of tyrosine bisphenolase activity in mushrooms	1.11 mM; 3.22 mM	Feng Y.-X. et al. (2021)
F1-a	NYRRE	Chinese quince seed	Inhibition of mushroom tyrosinase activity	8.69 mg/mL	Deng et al. (2020)
F1-b	RHAKF	Chinese quince seed	Inhibition of mushroom tyrosinase activity	1.15 mg/mL	Deng et al. (2020)

### 3.3 Melanin-synthesis-inhibiting peptides of mushrooms and bacteria

In a 1974 study, a peptide (Madhosingh and Sundberg, 1974) with tyrosinase inhibitory activity was first reported. Madhosnigh and Sundberg isolated two peptides from mushrooms. Naming them Ia and Ib. Their research revealed that Ia is a competitive inhibitor, while Ib is a non-competitive inhibitor. The main hydrolysis products of Ia were phenylalanine, aspartic acid, and glutamic acid in a 1: 1: 1 ratio. However, the peptide sequences of Ia and Ib remain unidentified.

Subsequently, Japanese researchers reported the first detailed sequence of a tyrosinase inhibitory peptide in their study on *Lactobacillus helveticus*. The cyclic peptide, Cycle [Pro-Tyr-Pro-Val], isolated from Swiss lactic acid bacteria, exhibited a tyrosinase IC50 of 1.5 mM, with inhibitory activity stronger than that of arbutin, which had an IC50 of 5.0 mM (Kawagishi et al., 1993) (Table 3).

**TABLE 3:** Melanin synthesis inhibitory peptides from mushrooms and bacteria.

Compound name	Peptide sequence	Source	Mechanism	IC50	References
Ia	Unknown	Mushroom	Competitive inhibition of mushroom tyrosinase in the presence of DOPA	Unknown	Madhosingh and Sundberg, (1974)
Ib	Unknown	Mushroom	Noncompetitive inhibition of mushroom tyrosinase in the presence of DOPA	Unknown	Madhosingh and Sundberg, (1974)
Unnamed	Cyclo [PYPV]	*Lactobacillus* helveticus	Inhibition of mushroom tyrosinase	1.5 mM	Kawagishi et al. (1993)

### 3.4 Non-natural peptides with anti-melanin synthesis activities

In an experiment conducted in Italy to develop nontoxic natural tyrosinase inhibitors, reversed-phase high-performance liquid chromatography (RP-HPLC) and ultraviolet detection were used to study the inhibitory effects of various dipeptides on DOPA pigment formation. The results showed that glycyl dipeptides (Gly-Asp, Gly-Lys, Gly-Phe, and Gly-Gly) inhibited melanin formation by directly inhibiting enzymes activity. In contrast, Gly-His inhibited subsequent reactions in converting dopaquinone to melanin. The authors also found that polyphenol oxidase could form binary complexes with substrates, inhibitors, or it can combine them to form ternary complexes. Among the peptides studied, Gly-His exhibited the strongest inhibitory effect (Girelli et al., 2004).

Xiao et al. constructed a pharmacophore model by molecular docking and group simulation to identify key functional groups in natural products that inhibited mushroom tyrosinase (Joompang et al., 2023). Several compounds were identified, including A5 and B16, which showed high tyrosinase inhibitory activity. A5 is similar to dipeptide WY, while B16 features a KFY structure, suggesting that C-terminal tyrosine residues play an important role in the tyrosinase inhibition. Eleven tripeptides derived from KFY were synthesized, with CRY and RCY exhibiting the highest tyrosinase inhibitory activities. The structures of CRY and RCY suggest that they can chelate with Cu ions in the active sites of tyrosinase, making them potential tyrosinase inhibitors.

## 4 Possible mechanisms of inhibiting melanin synthesis by bioactive peptides

### 4.1 Known mechanism of inhibiting melanin synthesis

#### 4.1.1 Inhibition of melanin synthesis by mediating oxidation pathway

Melanin plays a protective role in the human body. When ultraviolet rays irradiate the skin, ROS are generated (Chaiprasongsuk and Panich 2022; Yu et al. 2024). Excessive ROS can damage DNA, lipid peroxidation, and cause various issues including cancer (Bickers and Athar 2006; Lee et al., 2021; Chen et al., 2024). Studies have also shown that oxidative stress caused by UV radiation can contribute to hyperpigmentation (Sevilla et al., 2022). Grass Carp Scale Collagen Peptide (FTMGL) demonstrated antioxidant activity similar to kojic acid in B16F10 cells. FTMGL exerts its antioxidant activity by regulating glutathione (GSH) content and enhancing the levels of antioxidant enzymes (SOD, CAT, and GPx), which helps reduce the levels of superoxide dismutase (SOD) and malondialdehyde (MDA)- thus preventing pigment deposition caused by oxidative stress (Hu et al., 2022a). SOD and CAT are key antioxidant enzymes that maintain redox balance within cells (Pudlarz et al., 2020). The direct scavenging of free radicals and the recovery of antioxidant enzyme activity reduced by external stimuli are common antioxidant mechanisms by which peptides inhibit melanin synthesis (Errante et al., 2024).

It is worth mentioning that peptides with strong antioxidant activity often have strong anti-tyrosinase activity. There are abundant hydrophobic amino acids in bioactive peptides, such as Val, Ala, Gly, Iso, Leu, Phe and Pro, which have the ability to scavenge free radicals (Park and Jo, 2019). These amino acids also play an important role in anti-melanin synthesis (Ahuja et al., 2025). At the same time, aromatic amino acids (such as Trp, Leu, Phe, Tyr, Val, Ile) can stabilize active oxygen through direct electron transfer, and Tyr, Phe and Val can significantly enhance TIP's anti-tyrosinase activity (Nie et al., 2017; Shen et al., 2019; Joompang et al., 2020; Thaha et al., 2021; Aizawa and Yamamuro, 2024). Although the antioxidant and anti-melanin production pathways partially overlap, their specific molecular mechanisms have not been fully clarified. It is worth noting that there is still a lack of research on the antioxidant mechanism related to hyperpigmentation induced by environmental factors or endogenous factors (not ultraviolet radiation), which leads to the need to further explore the effectiveness and target of the targeted inhibitory peptide-based hyperpigmentation therapy strategy.

#### 4.1.2 Inhibition of melanin synthesis by mediating inflammatory reaction

Inflammation can also contribute to hyperpigmentation (Hossain et al., 2021). After treatment with substance P, an undecapeptide, the melanin content and tyrosinase activity were significantly downregulated in B16F10 mouse melanoma cells. Substance P may stimulate the phosphorylation of p70 S6K1 and inhibit the phosphorylation of p38 MAPK by activating the NK-1R receptor, thereby inhibiting TRP1 by MITF, thus playing an anti-melanin synthesis role (Ping et al., 2012). Later studies showed that calcitonin gene-related peptide (CGRP) at 500 ng/mL inhibited tyrosinase activity and melanin synthesis in B16F10 cells in a concentration-dependent manner, especially when combined with substance P (0.1–10 nm). However, CGRP alone did not affect melanin synthesis; its inhibitory effect was mediated by enhancing the expression of NK-1R (Zhou et al., 2015).

In addition to peptides, small molecules can also inhibit abnormal melanin deposition by mediating inflammatory reactions. Asiaticoside (MA) inhibits UV-induced hyperpigmentation by mediating the COX-2 and PGE pathways by inhibiting PAR-2 expression (Jung et al., 2013). Salvianolic acid [CA] has been shown to inhibit melanin deposition in zebrafish skin along with the production and transfer of melanin in skin cells. When CA and LP-GEL were applied together, skin wound healing accelerated, and inflammatory reaction and melanosis were inhibited (Su et al., 2024). Bay 11-7082 inhibits post-inflammatory pigmentation by suppressing inflammation and melanin production (Moon et al. 2025). Solamargine inhibits melanin synthesis in human skin cells induced by UVB by decreasing tyrosinase activity and the expression of MITF, TRP-1, and TRP-2. It also exerts an anti-inflammatory effect by modulating the MAPK/Nrf2/HO-1 signaling pathway (Zhao et al., 2022).

#### 4.1.3 Inhibition of melanin synthesis by mediating MITF-related pathway

It regulates the transcription of tyrosinase, tyrosinase-related protein 1 (TRP-1), and tyrosinase-related protein 2 (TRP-2), activates various target genes related to melanin synthesis, and influences the reproduction, proliferation, and survival of melanogenic cells (Wellbrock and Marais, 2005; Cheli et al., 2010; Vachtenheim and Borovansky, 2010). Peptides that inhibit melanin synthesis can modulate MITF expression through various pathways in order to inhibit melanin synthesis.

α-MSH binds to MC1R, which: increases cyclic adenosine monophosphate (cAMP) of the secondary messenger through adenylate cyclase (AC); stimulates protein kinase A(PKA) to translocate and phosphorylate CREB; activates its transcription activity, increases MITF expression, and then activates tyrosinase, promoting melanin synthesis (Pillaiyar et al., 2017). H89 can inhibit this process (Jian et al., 2011). PI3K/AKT/GSK3β pathway regulates melanogenesis by reducing MITF expression. CAMP inhibits PI3K, activates AKT, and increases GSK3β activity. After phosphorylation, it inhibits MITF from binding to a tyrosinase promoter and degrades it (Lee et al., 2021; Zhou et al., 2021). MEK/ERK/MITF Pathway, a negative pathway, and the ERK cascade reaction plays a role in cell growth, which can induce MITF phosphorylation and ubiquitination. The α-MSH trigger signal activates ERK after phosphorylation of MEK by cAMP, and p-ERK promotes MITF degradation and inhibits melanin production (Shirasugi et al., 2010; Huang et al., 2017). P38 MAPK is a member of MAPK, which can up-regulate MITF and tyrosinase. CAMP activates p38 MAPK, phosphorylates CREB and promotes MITF expression (Kang et al., 2020; Choi et al., 2022). SB203580 can interrupt p38 MAPK phosphorylation, and Fargesin inhibits melanin production through this pathway and the MEK/ERK/MITF pathway (Huang et al., 2016; Fu et al., 2019).

**FIGURE 2:**
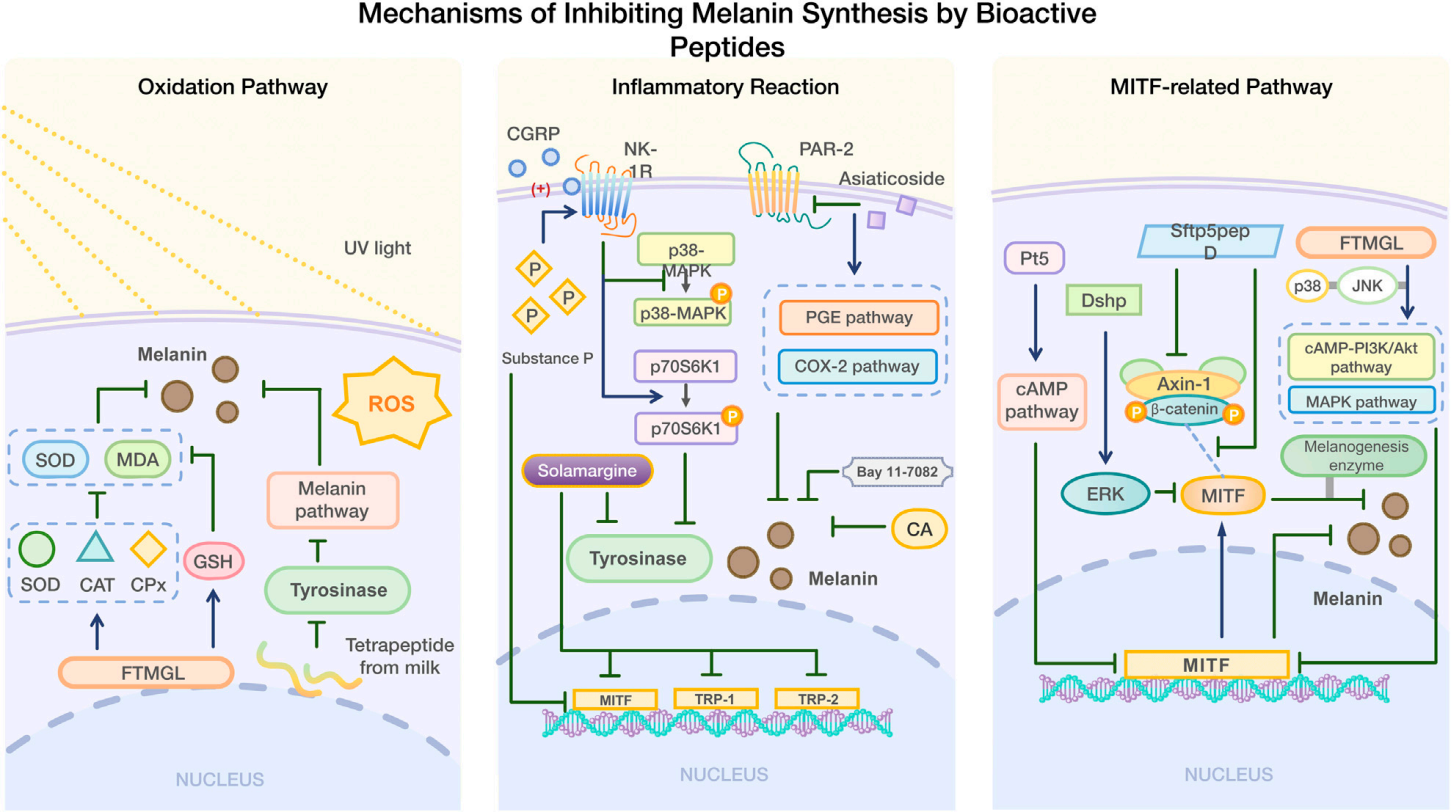
Mechanism of inhibiting melanin synthesis by bioactive peptides.

In addition to the above channels, there are some channels that are less studied. The zebrafish phosphopeptide Pt5 decreased MITF gene expression through the cAMP signaling pathway, inhibiting melanin synthesis (Liu et al., 2017). Dshp inhibits melanin synthesis by activating ERK and promoting MITF degradation (Choi et al., 2016). Sfrp5pepD disrupts the Wnt/β-catenin signaling pathway by inhibiting the interaction between Axin-1 and β-catenin, affecting the interaction between MITF and β-catenin. This reduces the expression of melanogenic enzymes and ultimately inhibits melanin synthesis (Choi et al., 2024). Additionally, FTMGL not only inhibits the pigment deposition through antioxidant activity, but also reduces MITF expression by modulating p38 and JNK in the cAMP-PI3K/Akt and MAPK signaling pathway, thereby inhibiting melanin synthesis in melanoma cells (Pudlarz et al., 2020) (Figure 2).

### 4.2 Characteristic analysis of melanin synthetic peptides

#### 4.2.1 Key sites of peptide inhibiting melanin synthesis

Peptides inhibit melanin synthesis, mainly by binding with TRP and MITF in melanin biological process. Molecular docking is a selective method used to help understand the drug-forming screening of small molecules such as peptides and macromolecules (Pinzi and Rastelli, 2019; Festa et al., 2020). This technique is used to predict how peptides bind to related targets that inhibit melanin synthesis, to further study its inhibition mechanism (Paggi et al., 2024).

The catalytic core of tyrosinase is composed of H_2_L_2_ tetramer, and its active center contains binuclear copper ions, which form a six-coordinated chelating structure through His61, His85, His94(CuA), His259, His263 and His296(CuB) (Baskaran et al., 2021). The redox cycle of Cu^2+^ is the key step to catalyze the oxidation of L-tyrosine to dopaquinone, which depends on the precise regulation of the hydrophobic environment in the active chamber (Bagherzadeh et al., 2015).

CuA and CuB are connected by an oxygen bridge to form a rigid structure, required for catalytic activity. Peptide inhibitors (such as VY-9) occupy hydrophobic pockets (His85, Phe264, etc.) near the cavity entrance, which prevent the substrate L-tyrosine from entering the active cavity and interfere with the coordination between Cu and His residues (such as the chelation of imidazole ring of His85 with Cu), resulting in the loss of enzyme activity (Baskaran et al., 2021; Sepehri et al., 2022; Sangtanoo et al., 2024). Even if the peptide does not directly bind to Cu, covering His residues (such as His259 and His263) will destroy the stable coordination of Cu and reduce its catalytic efficiency (Joompang et al., 2020). The entrance of the active cavity is composed of His85, Met280, Val283 and other residues, and its steric hindrance and π -π stacking (such as Phe264 and benzyl ring of the substrate) jointly determine the substrate selectivity (Yu and Fan, 2021). Short peptides are embedded in hydrophobic cavities in parallel through aromatic rings, enhancing π -π stacking, simulating the binding mode of natural substrates, and blocking the interaction between copper ion and His residues in hydrophobic cavities (Goldfeder et al., 2014).

Peptide inhibitors destroy tyrosinase function through a hydrogen bond network, hydrophobic interaction and electrostatic interaction, and their mechanisms can be divided into two categories: competitive inhibition and non-competitive inhibition. The aromatic amino acids of peptide (tyrosine) form hydrogen bonds with His263, simulating the interaction between phenolic hydroxyl groups of L-tyrosine and His263 (Goldfeder et al., 2014). The hydrophobic residue of the peptide is embedded in the hydrophobic pocket, which enhances the binding stability by van der Waals force and prevents the substrate from sliding into the active cavity (Hu et al., 2022b). Hydrophobic residues (such as Leu and Ala) cover the surface of the active cavity, which reduces the hydrophobic complementarity between the enzyme and the substrate, and further inhibits the catalytic efficiency (Hu et al., 2022b). The charged residues of peptide (such as Arg/Lys) form a salt bridge with the acidic residues on the enzyme surface (Glu256, Asp322), which induces the conformational change of the active center and destroys the stable coordination of copper ion (Deri et al., 2016).

**FIGURE 3:**
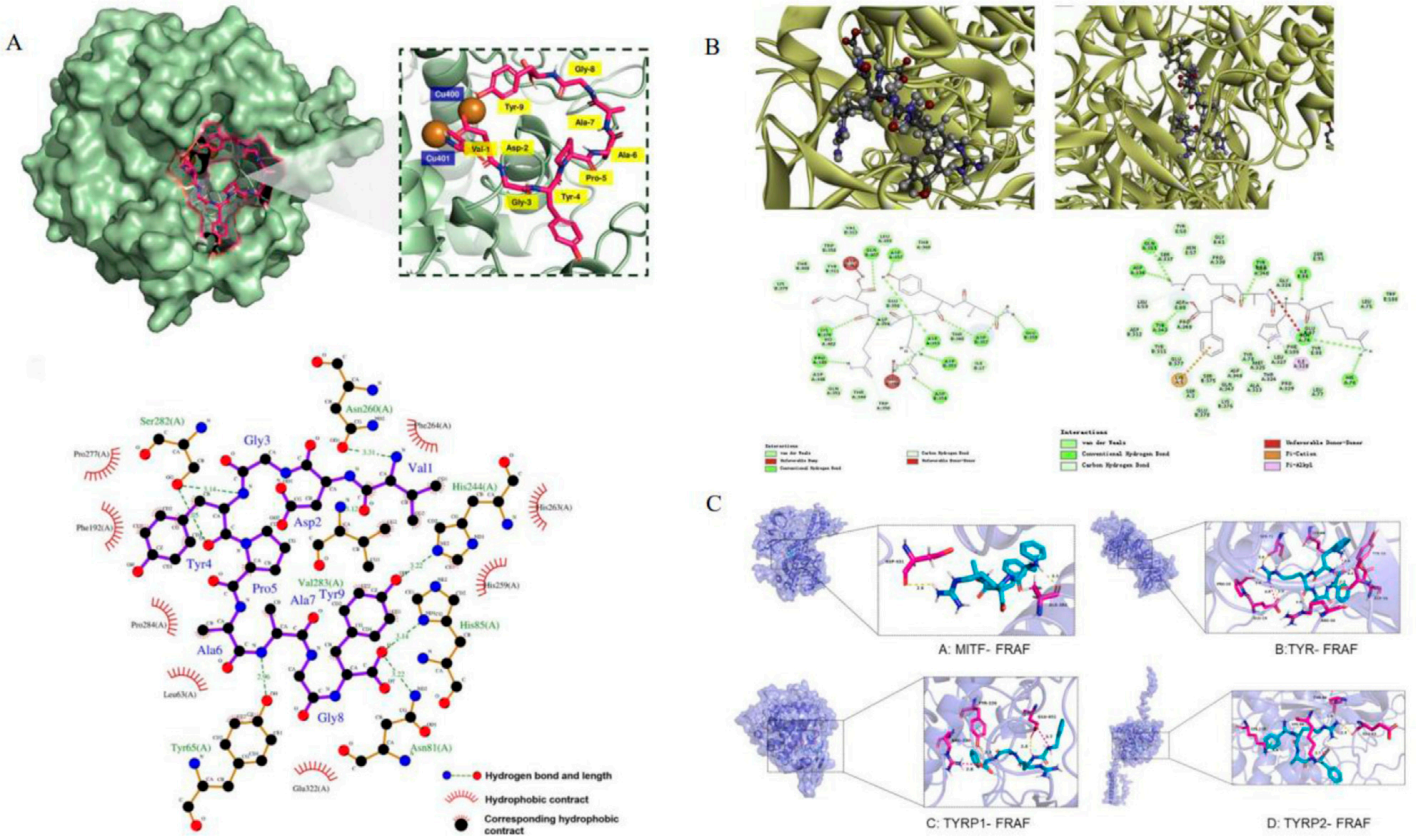
The figure shows the molecular docking diagram of tyrosinase and peptide, where **(A)** is the 3D and 2D docking model of VY-9 peptide and TYR (Sangtanoo et al., 2024), **(B)** contains multi-angle views: different conformations of the enzyme-peptide complex (including the docking model of NYRRE, RHAKF and TYR) are at the top Skin-care functions (Deng et al., 2020). **(C)** is the docking model of FRAF and TYR, MITF, TYR1 and TYR2 (Bai et al., 2024).

In addition, peptides (such as FRAF) form a multi-level inhibition network by targeting MITF, TYR and TYRP1/2, and its mechanism goes beyond single target intervention: FRAF forms hydrogen bonds with ASP431 and ALA384 of MITF, blocking the combination of MITF and target gene promoters (such as TYR and TYRP1) and inhibiting the expression of melanin synthesis-related enzymes. FRAF binds to PRO20 and GLU19 of TYR through hydrogen bonds, which interferes with its substrate binding pocket. It forms electrostatic interaction with GLU451 of TYRP1 and GLU63 of TYRP2, which destroys its auxiliary function of TYR and inhibits melanin transport and oxidation (Bai et al., 2024) (Table 4; Figure 3).

**TABLE 4:** Binding between peptides and MITF, TYR, TYRP1 and TYRP2 proteins.

Peptide	Combine object	Binding site	Binding energy (kcal/mol)	References
FRAF	MITF TYR TYR1 TYR2	hydrogen bonding hydrogen bonding and electrostatic interactions hydrogen bonding and electrostatic interactions hydrogen bonding, electrostatic interactions and hydrophobic interactions	−6.9 −9.1 −9.3 −8.9	Bai et al. (2024)
YYP	TYR	hydrogen bonding, electrostatic interactions and hydrophobic interactions	−7.6	Wang W. et al. (2023)
PYLK	TYR	hydrogen bonding	−6.9	Wang W. et al. (2023)
PHHF	TYR	hydrogen bonding	−7.3	Wang W. et al. (2023)
FRVK	TYR	hydrogen bonding	−7.0	Wang W. et al. (2023)
VDGYPAAGY	TYR	hydrogen bonding and hydrophobic interactions	−8.3	Sangtanoo et al. (2024)
YPNVY	TYR	Hydrogen bonding, electrostatic interactions and hydrophobic interactions	−10.4	Lin J. et al. (2024)

#### 4.2.2 Structure-activity relationship of melanin inhibitory peptides

**FIGURE 4:**
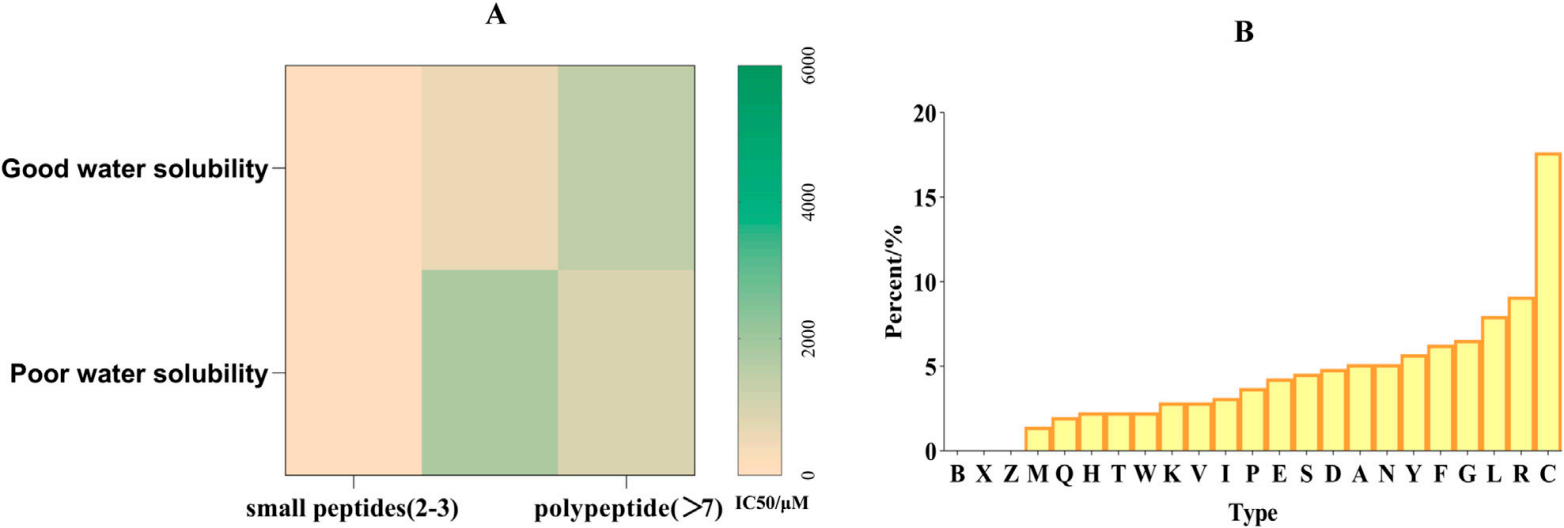
**(A)** Relationship between tyrosinase inhibitory ability of peptide and its length and hydrophilicity **(B)**. Proportion of amino acids in peptides with tyrosinase inhibitory activity.

The results show that the molecular weight, size and amino acid composition of peptide will significantly affect the anti-tyrosinase ability (Putri et al., 2025). Part A in Figure 4 shows that tyrosinase’s inhibitory ability of short peptides is higher than that of long peptides, and the peptides with good water solubility also have higher tyrosinase inhibitory activity. The result of polypeptides is just the opposite, because polypeptides are generally obtained by enzyme hydrolysis. Part B in Figure 4 shows that the cysteine content is as high as 17.66%, significantly higher than other amino acids. The reason for this may be that it can form disulfide bonds with tyrosinase to stabilize the peptide structure, participate in metal ion coordination, and maintain special conformation. The total amount of hydrophobic amino acids, including L (7.98%), I (3.13%) and V (2.85%), is relatively high, which may be related to the fact that they can form a hydrophobic core with tyrosinase or combine with hydrophobic pockets of tyrosinase. Figure 3B can provide some ideas for us to synthesize new tyrosinase inhibitory peptides. Usually, we will choose enzymes with better degrees of hydrolysis, so that the types of long peptides will be scarce, but too few quantities will lead to deviation in statistics. The cysteine content can be as high as 17.5%, which is significantly higher than other amino acids. The reason for this may be that it can form disulfide bonds with tyrosinase to stabilize peptide structure, participate in metal ion coordination and maintain special conformation. The inhibitory potential of peptides on tyrosinase is closely related to its amino acid composition and structural characteristics. For example, the C-terminal tyrosine residue is particularly critical because it significantly promotes tyrosine binding and inhibition by changing the conformation of the enzyme (Kose and Oncel, 2022). Similarly, tetrapeptides containing a N-terminal cysteine can exert tyrosinase inhibition by chelating with copper ions (Joompang et al., 2023). Basic residues (such as arginine) are paired with nonpolar amino acids (such as proline, alanine, valine and leucine), which have a strong inhibitory effect on tyrosinase (Zu et al., 2023). Many peptides exceed the optimal molecular weight threshold (500 Da) for skin penetration, and the partition coefficients (log P) are usually beyond the range required for effective absorption. This limits their ability to penetrate the skin, which will affect tyrosinase inhibition and skin delivery (Mortazavi and Moghimi, 2022). Peptides from fish scale gelatin and egg whites have proved that smaller peptides (400–600 Da) show higher copper ion chelating activity, highlighting the importance of molecular weights in peptide activity (Yap and Gan, 2020; Xue et al., 2022). In addition, studies have shown that the introduction of D-tyrosine into the N-terminal or C-terminal of pentapeptide-18 can reduce the melanin content by 50% at 500 μM, the tyrosinase activity by 18% at the N-terminal and by 25% at the C-terminal. Adding D-tyrosine can endow short functional cosmetic peptides with an anti-melanin effect, which is of great significance to the research of new cosmetics with whitening/anti-inflammatory and whitening/anti-aging functions (Ge et al., 2023).

Cyclization of peptides is a strategy that can make peptides have a more stable conformation, enhancing the stability of enzymatic proteolysis and improving the permeability through biological barriers (Karami et al., 2023). The cyclic peptide Massiliamide obtained from Gram-negative bacterium *Massilia albidiflava* DSM 17472T has an IC50 of 1.15 μM, and its tyrosinase inhibitory activity exceeds that of kojic acid and arbutin in positive control group (Frediansyah et al., 2021). New delivery systems, including liposomes, nanoparticles, nanoemulsions and microneedles, have been used to improve drug solubility, increase skin permeability or reduce skin irritation, thus enhancing the therapeutic effect (Jing-Yan et al., 2020; D'Souza and Shegokar, 2021; Xing et al., 2021; Saadh et al., 2024). The combination of these technologies and peptides will further increase the therapeutic effect of peptides.

## 5 Conclusion

Although natural and synthetic tyrosinase inhibitory peptides have made remarkable progress in the study of melanin regulation, there are still significant limitations in the existing achievements. The research on natural inhibitors from land and sea is mostly limited to the preliminary screening of known compounds, and their structure-activity relationship and mechanism of action have not been fully clarified - especially the targeting optimization of marine resources (only a small part of which have been verified) and human-specific tyrosinase (which has structural differences with mushroom sources) needs to be broken through. Structural biology research shows that the molecular weight, hydrophobic-hydrophilic balance and cyclization strategy of peptide are very important to the inhibitory effect of TYR. The synergistic effect of aromatic residues, arginine and negatively charged histidine can enhance the stability of enzyme binding, and the regulation of hydrophobic N-terminal residues on aging-related enzyme activities reveals a new optimization direction.

However, the clinical transformation of natural peptides is limited by stability and delivery efficiency, while synthetic peptides need to solve the problems of species-specific differences and long-term safety verification. In the future, we need to focus on three major directions: ① establishing a multi-dimensional screening platform (such as 3D melanocyte models) to accelerate the rational design of humanized peptides; ② Developing a cyclic peptide-liposome co-delivery system to break through the skin barrier and enhance targeting; ③ Identify the regulatory nodes of TYR in melanoma signal pathway through organ-like and preclinical studies, and promote the transformation from basic research to therapeutic application. The collaborative innovation of natural and synthetic peptides will provide a therapeutic paradigm with both efficacy and safety in this field.
